# Dictionary of Practical Medicine

**Published:** 1846-07

**Authors:** 


### DICTIONARY OF PRACTICAL MEDICINE.

The 14th part of this able publication has reached our bookstores. Its matter is elaborate and rich, insanity, inflammation of the intestines, irritation, itch, and the diseases of the kidneys being among the subjects discussed in it.

